# Dual Requirement of Cytokine and Activation Receptor Triggering for Cytotoxic Control of Murine Cytomegalovirus by NK Cells

**DOI:** 10.1371/journal.ppat.1005323

**Published:** 2015-12-31

**Authors:** Bijal A. Parikh, Sytse J. Piersma, Melissa A. Pak-Wittel, Liping Yang, Robert D. Schreiber, Wayne M. Yokoyama

**Affiliations:** 1 Department of Pathology and Immunology, Washington University School of Medicine, St. Louis, Missouri, United States of America; 2 Division of Rheumatology, Department of Medicine, Washington University School of Medicine, St. Louis, Missouri, United States of America; 3 Howard Hughes Medical Institute, Washington University School of Medicine, St. Louis, Missouri, United States of America; Emory Vaccine Center, UNITED STATES

## Abstract

Natural killer (NK) cells play a critical role in controlling murine cytomegalovirus (MCMV) and can mediate both cytokine production and direct cytotoxicity. The NK cell activation receptor, Ly49H, is responsible for genetic resistance to MCMV in C57BL/6 mice. Recognition of the viral m157 protein by Ly49H is sufficient for effective control of MCMV infection. Additionally, during the host response to infection, distinct immune and non-immune cells elaborate a variety of pleiotropic cytokines which have the potential to impact viral pathogenesis, NK cells, and other immune functions, both directly and indirectly. While the effects of various immune deficiencies have been examined for general antiviral phenotypes, their direct effects on Ly49H-dependent MCMV control are poorly understood. To specifically interrogate Ly49H-dependent functions, herein we employed an *in vivo* viral competition approach to show Ly49H-dependent MCMV control is specifically mediated through cytotoxicity but not IFNγ production. Whereas m157 induced Ly49H-dependent degranulation, efficient cytotoxicity also required either IL-12 or type I interferon (IFN-I) which acted directly on NK cells to produce granzyme B. These studies demonstrate that both of these distinct NK cell-intrinsic mechanisms are integrated for optimal viral control by NK cells.

## Author Summary

Natural killer (NK) cells play a crucial role in the protection of the host against viruses and in particular herpesvirus infections. Through their activation receptors which recognize surface ligands on target cells, NK cells can mediate direct killing (cytotoxicity) of virus-infected cells and produce their signature cytokine IFNγ, but it is unclear to what extent these effector arms contribute to clearance of murine cytomegalovirus (MCMV) infections. Additionally, NK cells are activated through their cytokine receptors but the interplay between the activation and cytokine receptor pathways has not been elucidated. Herein we devised a viral competition assay that allowed direct evaluation of the requirements for NK cell mediated MCMV control. We found that cytotoxicity is the main effector mechanism by which NK cells control virus infection through activation receptors. Complemented by *in vitro* assays, we delineated the requirements for NK cell cytotoxicity and identified a 2-step mechanism for NK-mediated cytotoxicity. Firstly, NK cells require cytokine signals for the accumulation of cytotolytic proteins. Secondly, direct target cell recognition results in release of the cytolytic cargo and lysis of virus-infected cells. Our study demonstrates the integration of NK activation and cytokine receptor signals are required for effective viral control.

## Introduction

Natural killer (NK) cells are a critical component of the innate immune system. They play essential roles in controlling viral infections as illustrated in patients with selective NK cell defects who are susceptible to recurrent herpesvirus infections [1]. These clinical responses are recapitulated in animal studies, particularly with murine cytomegalovirus (MCMV), a natural mouse pathogen of the β-herpesvirus family, thus allowing further mechanistic insight. In the C57BL/6 (B6) mouse strain, NK cell-mediated control of MCMV infection is dependent upon the Ly49H activation receptor which is responsible for genetic resistance and is absent in susceptible strains such as BALB/c [2–4]. Ly49H specifically recognizes the MCMV-encoded ligand, m157, triggering NK cell activation and subsequent control of MCMV [5, 6]. Ly49H associates with the DAP12 adaptor molecule required for Ly49H surface expression and signaling. DAP12 has cytoplasmic immunoreceptor tyrosine-based activation motifs (ITAM) and directly mediates Ly49H signaling [5–7]. While the requirement of the related adapter molecule DAP10 is controversial [8, 9], Ly49H-dependent antiviral control is also illustrated by selection pressure in T cell-deficient hosts in which escape viral clones deficient in m157 expression emerge after several weeks following infection [10]. Unlike with the wild-type (WT) virus, these escape MCMV clones cannot be controlled by NK cells, even in Ly49H-sufficient mouse strains [10, 11]. Recently, infection with multiple, purified wild isolates of MCMV confirmed that Ly49H^+^ NK cells could only control m157-sufficient virus, resulting in an apparent outgrowth of m157-deficient strains [12]. Thus, Ly49H-m157 interactions are critical for MCMV control.

As with other NK cell activation receptors, Ly49H recognition of m157 *in vitro* can trigger two major effector functions: target-cell lysis (cytotoxicity) and cytokine production [5, 13]. Indeed, NK cell activation receptor ligands expressed on insect cells are sufficient to trigger NK cell degranulation as measured by cell-surface CD107a (LAMP1) staining [14]. Stimulation of NK cells with plate-bound anti-activation receptor antibodies, such as anti-Ly49H, causes similar NK cell activation and target killing [13, 15]. In addition, Ly49H-dependent stimulation *in vitro* leads to release of the signature NK cell cytokine, interferon gamma (IFNγ) [5], which has direct antiviral activity and can modulate subsequent immune responses [16, 17]. Indeed, prior to identification of the role of Ly49H, NK cell-dependent control of MCMV in B6 mice was reported to be dependent on both cytotoxicity and IFNγ [18, 19]. A more recent report also supports a role for IFNγ in NK cell control of MCMV but cytotoxicity was not examined [20]. Moreover, NK cells also release chemokines upon Ly49H stimulation [21]. Thus, it is still unclear which NK cell effector mechanisms, i.e. cytotoxicity versus cytokine/chemokine production, contribute specifically to Ly49H-dependent clearance of MCMV.

In addition to stimulation through their activation receptors, NK cells can be non-specifically activated through cytokine receptors to produce other cytokines [22–24]. During MCMV infections, other immune and non-immune cells produce an array of pro-inflammatory cytokines including IFNα/β [25] (IFN-I), IL-12 and IL-18 [26]. Many of these cytokines are induced by pattern receptors, such as TLRs, which are required for MCMV control, even in Ly49H-sufficient mice [27]. Importantly, these cytokines can directly or indirectly stimulate NK cells to produce IFNγ and this early production of IFNγ by NK cells can influence antiviral responses. However, the pleiotropic nature of the antiviral cytokines, such as IFN-I, IL-12, and IL-18, makes it difficult to dissect their broader effects on viral replication from their role in stimulating NK cell cytokine production [16].

Indeed, several cytokines are also capable of potentiating NK cell cytotoxic function [24]. Pretreatment of mice with poly(I:C) enhances both *in vivo* and *ex vivo* cytotoxic responses against various NK cell targets [28]. This effect is due to indirect stimulation of NK cells via accessory cells responding to poly(I:C) through the nucleic acid sensors, subsequently inducing the production of IL-12 and IFN-I [29]. These cytokines also induce DCs to produce IL-15, which has been linked to “priming” of NK cells thereby enhancing killing of target cells as well as increasing production of IFNγ and granzyme B (GzmB) [30]. In addition, resting murine NK cells express abundant transcripts for their cytotoxic proteins; cytokines such as IL-2 and IL-15 induce production of GzmB and perforin protein, which also correlates with increased target cell lysis [31]. It has been postulated that the elevated protein levels of these effector molecules facilitate NK cytotoxicity mediated through activation receptors, such as Ly49H, but direct evidence is not available and the roles of other cytokines have not been explored.

Therefore, it has been challenging to determine the precise NK cell effector mechanism mediated by Ly49H-dependent activation of NK cells in controlling MCMV as well as the role of non-specific cytokine stimulation of NK cells in this control. Herein we employed an *in vivo* viral competition approach which, together with *in vitro* and other *in vivo* studies, strongly suggests that NK cells require triggering of the Ly49H activation receptor for degranulation and that certain cytokine signals are also required to promote sufficient GzmB protein levels for effective cytotoxic elimination of MCMV.

## Results

### Characterization of global immune defects on splenic MCMV titers

**Fig 1:**
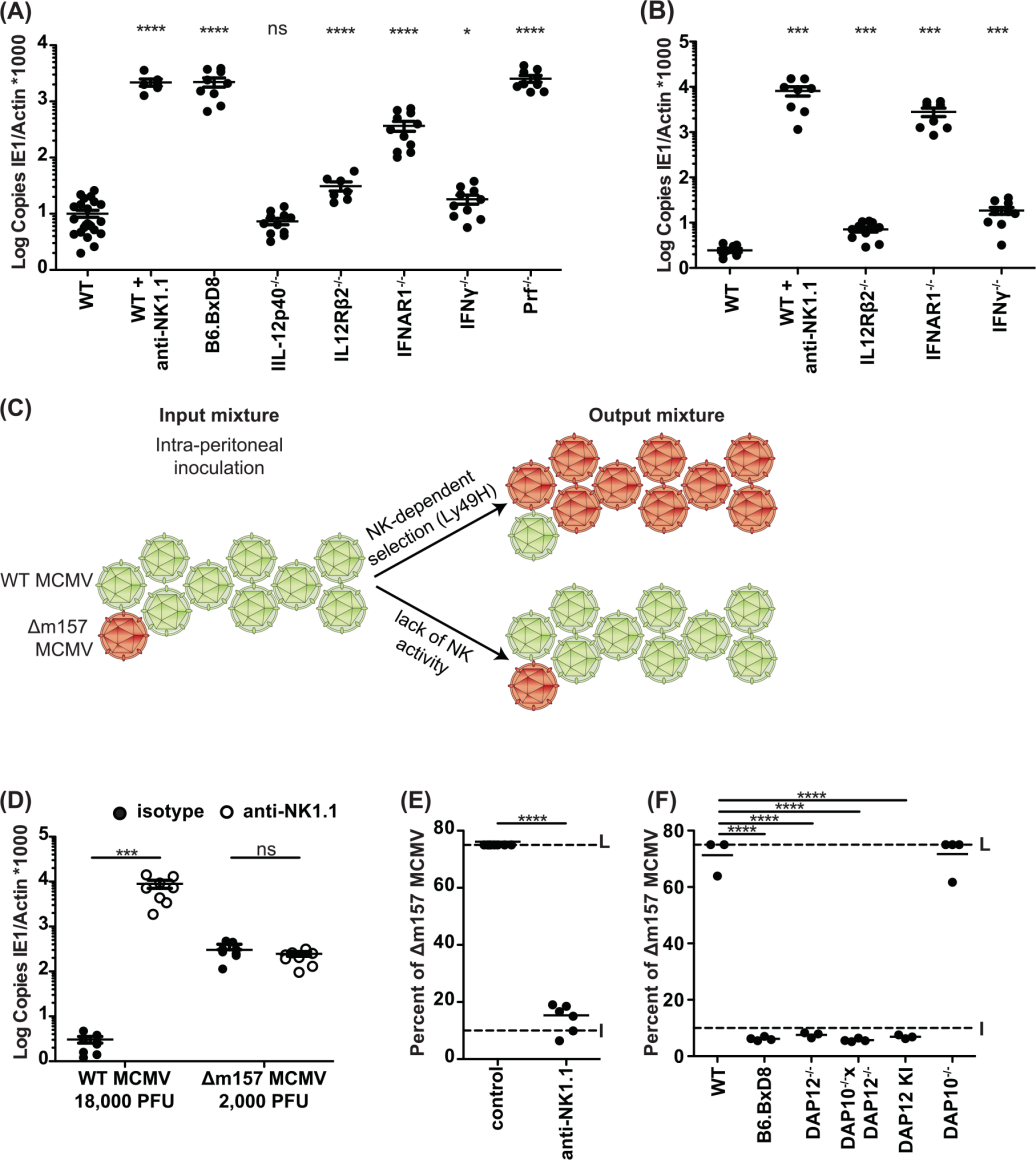
NK cells specifically control MCMV infection via cytotoxic effector molecules. **(A)** Splenic viral titers were analyzed by qPCR following a three-day infection with WT MCMV (5000 PFU/mouse) or **(B)** five-day infection with WT MCMV (20000 PFU/mouse). Statistical analysis performed by comparing values from WT mice with genetically modified or antibody-treated mice. **(C)** Schematic of the dual-infection system depicting the starting viral mixture and two possible results: Ly49H-dependent control of WT MCMV with resulting selection of Δm157 clones *vs*. equivalent replication of both MCMV strains due to absence of Ly49H-dependent control of WT MCMV. **(D)** Splenic viral titers following a five-day infection, as in **(B)**, with the indicated dose and strains following antibody treatment. **(E-F)** Splenic viral escape (Δm157) frequency determined five days after a mixed MCMV infection with WT MCMV and Δm157 (20000 PFU/mouse). NK1.1 antibody was administered where indicated 48hrs prior to infection. Data are combined from two independent experiments with individual points representing a single mouse. L = Limit of quantification. I = Input frequency (10% frequency of Δm157). ****p < .0001, ***p < .001, **p < .01, *p < .05, ns = not significant.

To address the relative impact of NK cells and Ly49H versus cytokine signaling on MCMV infection, we evaluated the splenic titers of mice three days after challenge with a low dose (5000 PFU) of a wild type (WT) MCMV clone (Fig 1A). Importantly, our studies allowed a direct comparison of different genetically deficient mice on the C57BL/6 (B6) background. We found the expected marked susceptibility in B6 mice depleted of NK1.1-expressing cells or that were genetically deficient in Ly49H expression (B6.BxD8 strain). Compared to B6 mice, we also noted either no effect of IL-12 cytokine deficiency (p40 subunit) or relatively minor effects of IL-12Rβ2 (three-fold) and IFNγ (two-fold) deficiency on titers. Moreover, interferon α/β receptor 1 deficiency (IFNAR1^-/-^) increased titers over 30-fold but not to the levels seen with NK cell or Ly49H deficiency. The loss of perforin (Prf1^-/-^) resulted in elevated viral titers similar to both Ly49H and NK cell deficiency, suggesting that cytotoxic effector function is a key mechanism in early splenic control of MCMV, but the role of cytotoxic T cells could not be excluded. On the other hand, when we challenged a cohort of mice for five days with a larger dose (20000 PFU) of MCMV, we observed somewhat different effects (Fig 1B). While we again observed the expected susceptibility in mice depleted of NK1.1-expressing cells and also a minor effect of IL-12Rβ2 and IFNγ deficiency, IFNAR1^-/-^ mice demonstrated markedly increased titers (over 1000-fold) approaching levels seen with NK cell depletion (Fig 1B). Thus, overall viral titers suggest dominant roles for perforin and IFN-I in control of MCMV with contributions from IL-12 and IFNγ, but their relationship to Ly49H-dependent responses remained unclear as their effects can be dependent on the viral dose.

### *In vivo* viral competition assay to determine Ly49H-dependent NK cell activity against MCMV

Since it was challenging to attribute functional defects specifically to NK cells and Ly49H when genetically deficient mice were infected with WT MCMV and viral titers or survival were assessed, we designed and validated an *in vivo* dual-infection, viral competition assay. To specifically determine the role of Ly49H-dependent and independent control of MCMV *in vivo*, we inoculated mice with a mixture of MCMV lacking m157 expression (Δm157) due to a single point mutation, and WT MCMV at a defined frequency. We hypothesized that Ly49H-dependent control of WT MCMV should result in a proportional increase in Δm157 virions, as measured by the splenic frequency of Δm157 relative to WT MCMV (Fig 1C). By contrast, absence of Ly49H-dependent control should not lead to selection of Δm157.

To validate this approach, we first studied B6 mice individually infected with WT MCMV or Δm157 and treated with either isotype control antibody or anti-NK1.1 (Fig 1D). The dose of viral inoculum was chosen to reflect the frequencies to be used in the dual-infection assay (Fig 1C). We found that depletion of NK cells led to markedly elevated WT MCMV levels with no additional effect on Δm157 titers which were already elevated in control antibody-treated mice despite a lower inoculum (Fig 1D). We next characterized the splenic frequency of Δm157 virus in NK cell-depleted and control mice after inoculation with a mixture of 90% WT MCMV and 10% Δm157 viruses (Fig 1E). Indeed, in B6 mice treated with the control antibody, the Δm157 viral frequency increased from input level (10%) to the limit of the assay (75%), indicating selective outgrowth of Δm157. However, in mice depleted of NK cells, the Δm157 MCMV frequency was similar to the input inoculum (Fig 1E), even though overall viral titers were significantly elevated (S1A Fig). These results demonstrate that without the selective pressure contributed by NK cells, both WT and m157-deficient viruses replicate to similar levels *in vivo*.

The degree of viral burden might contribute to differences in the ability of NK cells to clear WT MCMV during the viral competition assay. Consistent with this, we have seen that splenic selection of Δm157 in B6 mice can be overwhelmed with a larger viral inoculum (S1B and S1C Fig). However, 1e6 PFU per mouse (50-fold higher than in the experiments performed within the rest of this study) were required to observe such an effect. Therefore, we utilized a low inoculum (2e4 PFU per mouse) that allowed for optimal discrimination of Ly49H-dependent clearance without overwhelming NK cell-dependent viral control.

When we infected the Ly49H-deficient B6.BxD8 strain, we observed no Δm157 selection, demonstrating that the co-infection reflected Ly49H-dependent antiviral function (Fig 1F). There was also impaired Δm157 selection in DAP12^–/–^and DAP10^–/–^DAP12^–/–^mice. As genetic deficiency of DAP12 prevents surface expression of Ly49H, we also analyzed DAP12 knock-in (KI) mice having a mutation in the ITAM that allows normal Ly49H surface expression with defective signaling [7]. The DAP12 KI mice also showed no selection. Although DAP10 can associate with Ly49H, the single DAP10^–/–^animals allowed Δm157 to accumulate, indicating that DAP10 is not required as previously reported [9] while contrasting with other reports [8]. Consistent with previous data (Figs 1E and S1A), viral titers were elevated in all dual-infected mice in which selection was impaired (S1D Fig). Thus, Ly49H and DAP12 are required for Δm157 selection, consistent with previous studies on Ly49H-DAP12-dependent mechanism for WT MCMV control [5, 7] and emergence of Δm157 in T cell-deficient mice [10].

### Δm157 selection is preserved during individual deficiencies affecting innate immunity

**Fig 2:**
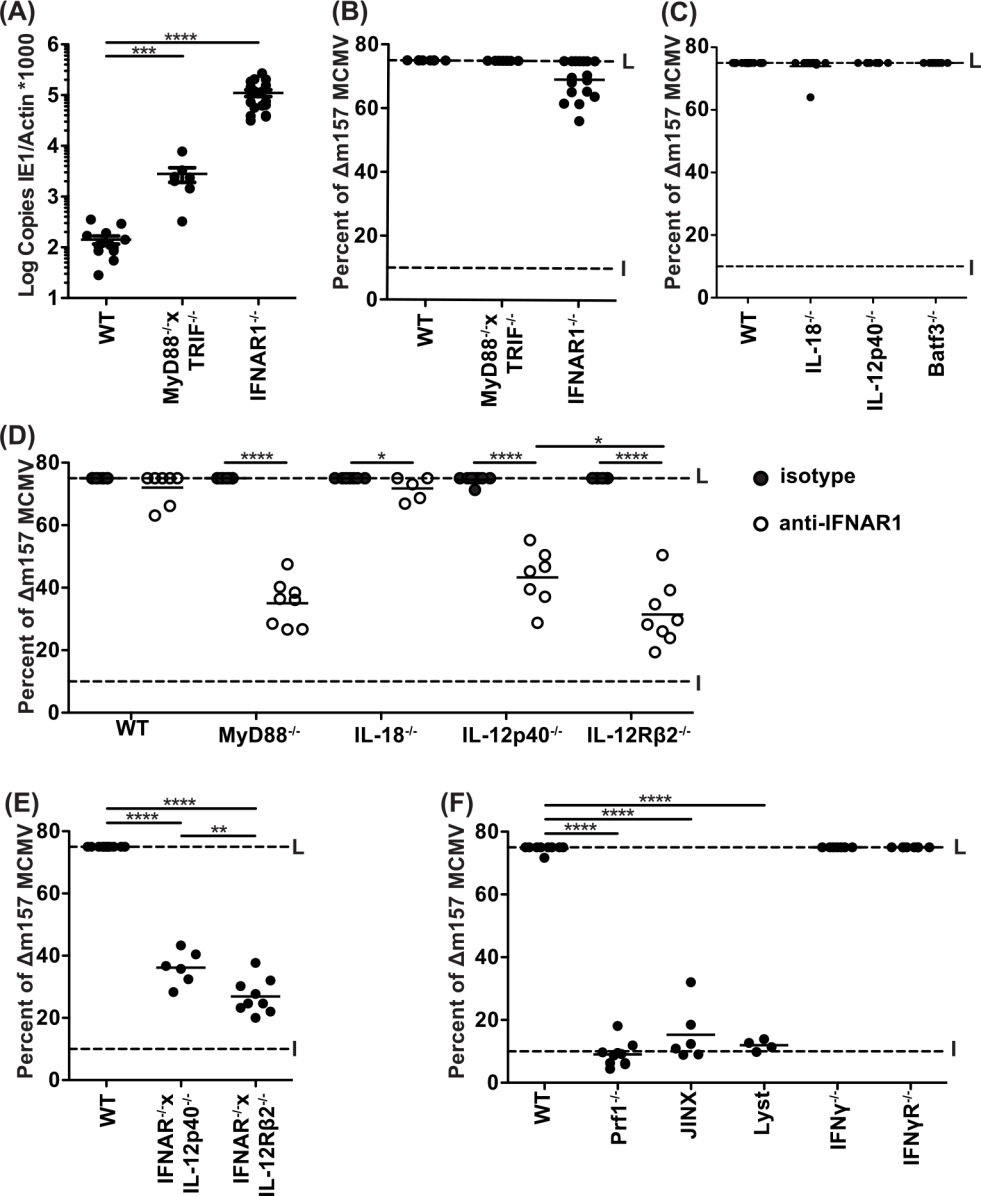
NK cell cytotoxic control of MCMV requires both IL-12 and IFN-I signaling. (A) Splenic viral titers and (B-F) escape (Δm157) frequency determined five days after a mixed MCMV infection (20000 PFU/mouse), as in Fig 1. (D) Anti-IFNAR1 antibody (or isotype control) was administered where indicated 24hrs prior to infection. Data are combined from two to three independent experiments with individual points representing a single mouse. L = Limit of quantification. I = Input frequency (10%).

When inoculated with a mixed MCMV inoculum, mice with TLR signaling deficiency due to absence of both TLR signaling adapters (myeloid differentiation factor 88, MyD88; and Toll/IL-1R domain-containing adapter inducing IFN-β, TRIF) demonstrated enhanced susceptibility (Fig 2A). Though consistent with prior reports demonstrating that TLR deficiency leads to enhanced susceptibility to MCMV [27], MyD88^–/–^TRIF^–/–^mice showed no defect in Δm157 selection (Fig 2B), suggesting that the TLR signaling-dependent results were not due to effects on Ly49H-dependent control. Similarly, we observed that deficiency of IFNAR1 markedly affected infection with a mixed MCMV inoculum (Fig 2A) but did not markedly affect Δm157 selection (Fig 2B). Notably, in both cases, there was Δm157 selection even though overall viral titers were elevated in the absence of TLR or IFN-I responses (Fig 2A), indicating that the viral competition assay could reveal Ly49H-dependent effects even during marked viremia.

We next assessed the role of two other cytokines, IL-18 and IL-12, previously demonstrated to enhance NK cell function both *in vivo* and *in vitro* [32]. However, deficiency of IL-18 or IL-12p40 also did not result in loss of viral selection (Fig 2C). Furthermore, Batf3-dependent conventional DC subsets that present IL-15 to NK cells were also not required to control WT MCMV (Fig 2C). Consistent with the lack of Δm157 outgrowth, viral titers were also similar to B6 mice (S1E Fig). Taken together, these findings demonstrate that Δm157 selection remains intact in the face of individual deficiencies of TLR signaling, IFN-I, IL-18, or IL-12, as well as Batf3 deficiency. Since overall viral titers were higher in the absence of TLR signaling and IFN-I deficiency, our findings suggest that Ly49H-dependent control of WT MCMV could occur normally despite global deficits of innate antiviral immunity which affect Ly49H-independent control of both WT MCMV and Δm157.

### Δm157 selection is impaired in the absence of both IFN-I and IL-12 signaling

Since we found no major effect on Δm157 selection in mice with single immune deficiencies, we next assessed their potential redundant roles with IFN-I. The combination of anti-IFNAR1 blockade with MyD88 deficiency markedly impaired Δm157 selection (Fig 2D). Notably, this loss of selection was not seen in B6 mice treated with anti-IFNAR1 or MyD88^–/–^mice treated with an isotype control, indicating that both MyD88-dependent and IFN I-dependent pathways are critical for Δm157 selection by Ly49H^+^ NK cells. Since MyD88^–/–^mice fail to elaborate both IL-12 [27] and IL-18 [33], we next combined IFNAR1 blockade with IL-12 or IL-18 deficiency. While IFNAR1 blockade in IL-18^–/–^mice had no effect on Δm157 selection, IFNAR1 blockade plus either IL-12p40 or IL-12Rβ2 deficiency led to impaired selection, similar to IFNAR1 blockade in MyD88-deficient mice (Fig 2D). To further substantiate these findings, we assessed IFNAR1^–/–^mice genetically deficient in either IL-12 cytokine or receptor (Fig 2E). Both of these compound cytokine genetic deficiencies compromised Δm157 selection and resulted in elevated viral titers (S1F Fig). Taken together, these findings support a redundant relationship between IL-12 and IFN-I in promoting Ly49H-dependent control of WT MCMV.

### Δm157 selection in the spleen is dependent upon cytotoxic degranulation but not IFNγ

To better understand how IL-12 and IFN-I affect Ly49H^+^ NK cell activities, we studied the classical NK cell effector functions, cytotoxicity and IFNγ production. Perforin was critical for promoting Δm157 selection (Fig 2F). Similarly, Jinx [34] and Lyst [35] mice, both of which have distinct defects in cytotoxicity, recapitulated the impaired Δm157 selection observed in Prf1^–/–^mice. Surprisingly, however, Δm157 selection was observed in IFNγ^-/-^ and IFNγR^-/-^ mice; both were similar to B6. Viral titers supported our observations in these strains (S1G Fig). However, a prior publication has demonstrated that cytotoxic control of MCMV during acute infection occurs primarily within the spleen and IFNγ-dependent control is more prominent in the liver [19] while a more recent study suggests IFNγ affects both spleen and liver control [18]. To address whether IFNγ contributes to m157-dependent control in the liver, we evaluated the consequences of hepatic MCMV selection in B6, IFNγ^-/-^, and Prf1^-/-^ mice (S1H Fig). We found that Δm157 selection in the liver was incomplete in the B6 background, confirming that Ly49H-dependent mechanisms are not as prominent in the liver compared to the spleen. As observed in the spleen (Fig 2F), Prf1^-/-^ mice were not able to select for Δm157 in the liver (S1H Fig), demonstrating that the primary mechanism responsible for selection in these organs requires perforin-mediated cytotoxicity. We observed only partial hepatic Δm157 selection in IFNγ^-/-^ mice, suggesting that in contrast to the spleen, IFNγ exerts a mild influence on MCMV control in the liver. The hepatic titers were mildly elevated (about 10-fold) in the IFNγ^-/-^ strain and markedly higher (1000-fold) in Prf1^-/-^ mice when compared to B6 mice (S1I Fig), similar with what we observed in the spleen (S1G Fig). In summary, these results suggest that Ly49H-dependent control of WT MCMV in the spleen and liver depends on perforin-mediated cytotoxicity and IFNγ partially contributes to Δm157 selection in the liver.

### Signaling through Ly49H alone stimulates degranulation

**Fig 3:**
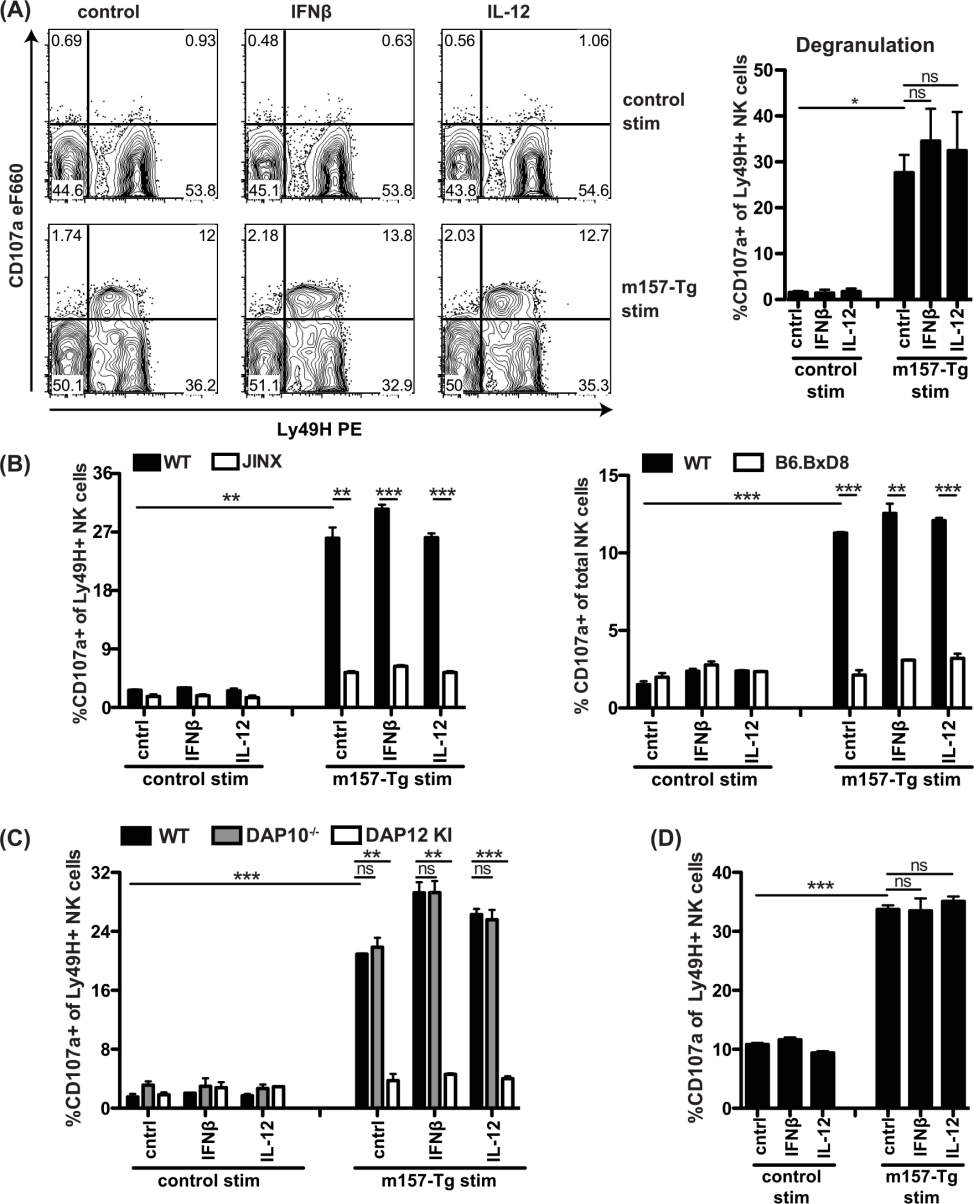
*In vitro* stimulation of NK cells with m157-Tg splenocytes alone induces DAP12-dependent degranulation. (A-C) Splenocytes from CD45.2 WT or indicated strain were mixed with CD45.1/2 congenic m157-Tg or WT splenocytes at a 1:1 ratio in the presence of the indicated cytokines. IFNβ was used at 100–200 U/ml, IL-12 was used at 10–20 ng/ml. NK cell degranulation was measured after a 6–9 hr incubation. (A) Representative FACS plots are shown on the left and percentage of CD107a^+^ cells within the Ly49H^+^ NK cell population of biological duplicates is shown on the right. (B) Jinx or B6.BxD8 splenocytes were mixed with m157-Tg or control splenocytes. For experiments with B6.BxD8 percentage of CD107a^+^ NK cells is calculated over the whole NK cell population. (C) DAP10^-/-^ or DAP12 KI splenocytes were mixed with adapter-expressing m157-Tg or control splenocytes. (D) Purified NK cells were cultured with m157-Tg or WT control murine embryonic fibroblasts (MEF) and analyzed as in (A). Data are representative of at least two independent experiments.

To further evaluate the role of IL-12 and IFN-I in Ly49H-dependent MCMV control, we performed *in vitro* assays. As cytotoxicity is required for Ly49H-dependent MCMV control, we first studied NK cell degranulation. NK cells did not degranulate in response to cytokines alone (Fig 3A). Instead, stimulation with transgenic m157-expressing (m157-Tg) splenocytes [36] but not WT splenocytes induced degranulation in Ly49H^+^ cells. Almost all NK cells that expressed lower levels of cell-surface Ly49H (Ly49H^dim^) were CD107a^+^, suggesting that virtually all Ly49H^+^ NK cells that encountered m157 during the course of the experiment subsequently degranulated. The CD107a-specific response to m157-Tg stimulators was abrogated in Jinx and B6.BxD8 mice, confirming that Ly49H-signaling specifically induced degranulation (Fig 3B). Jinx NK cells displayed the Ly49H^dim^ population after m157-Tg exposure, indicating that these NK cells were indeed triggered but did not degranulate. Stimulation of DAP12-deficient NK cells did not induce any specific degranulation whereas stimulation of DAP10-deficient NK cells resulted in degranulation similar to WT controls (Fig 3C), indicating that DAP12, but not DAP10 is involved in Ly49H-mediated degranulation and consistent with our *in vivo* data on Δm157 selection (Fig 1F). Interestingly, m157-Tg stimulation did not induce the Ly49H^dim^ population in DAP12-deficient NK cells, indicating that signaling through Ly49H and DAP12 is required for the emergence of this population. These data also show that DAP12 is cell-intrinsically required on NK cells, which was also confirmed by co-culture of purified NK cells with m157-Tg murine embryonic fibroblasts (MEF) (Fig 3D). Finally, combining m157-Tg stimulation with cytokines did not further enhance the percentage or MFI of the CD107a^+^ NK cells (Fig 3A–3D). Together, these *in vitro* data show that activation receptor engagement by ligand alone is sufficient for degranulation, and suggest that IL-12 and IFN-I play another role to enhance NK cell cytotoxicity.

### Signaling through cytokine receptor is solely required for GzmB production in NK cells

**Fig 4:**
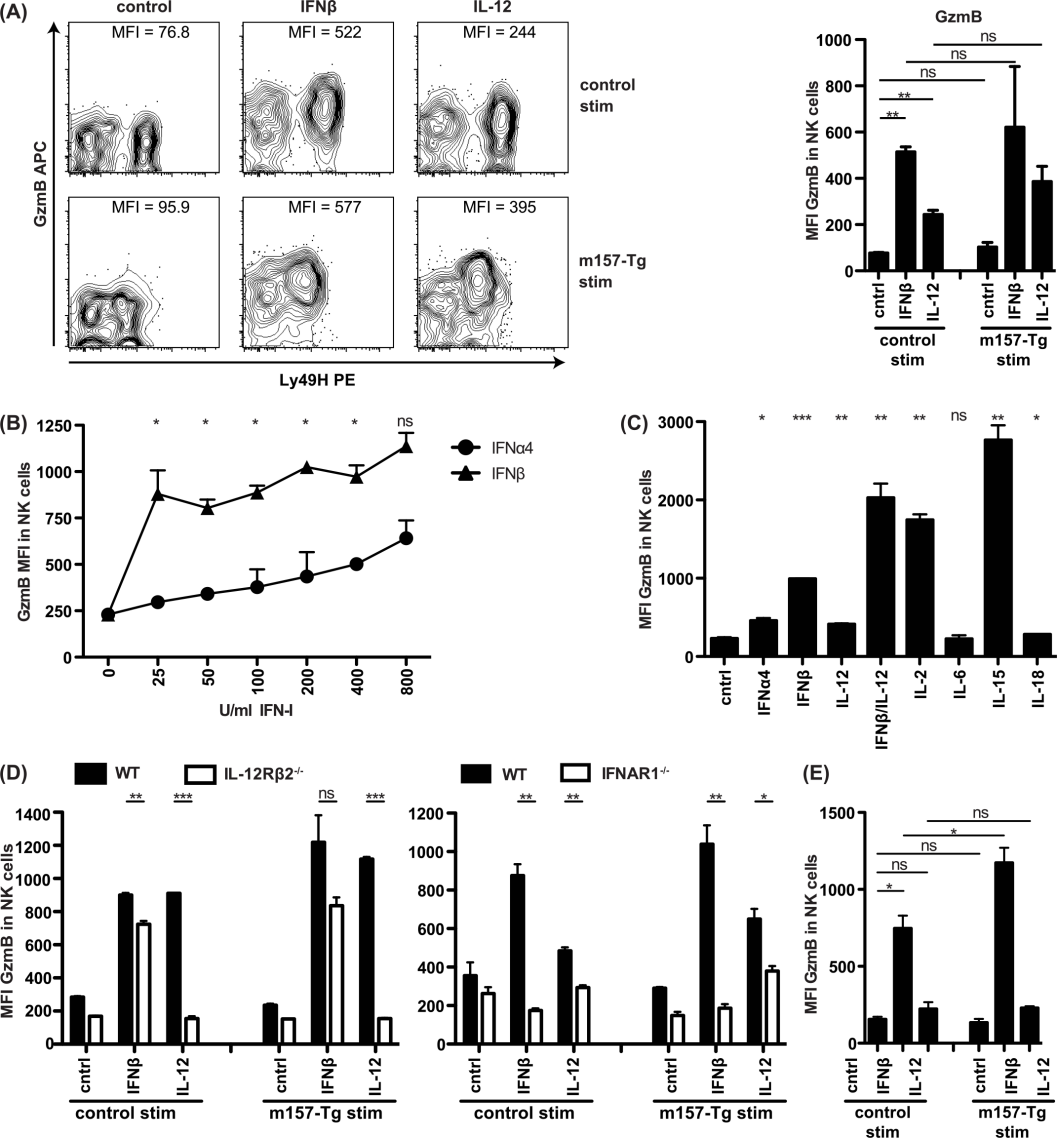
*In vitro* stimulation of NK cells with IFN-I and IL-12 induces GzmB expression in a cell-intrinsic manner. (A-D) Splenocytes from CD45.2 WT or indicated strain were mixed with CD45.1/2 congenic m157-Tg or WT splenocytes in the presence of the indicated cytokines. IFNβ was used at 100–200 U/ml, IL-12 was used at 10–20 ng/ml, unless otherwise indicated. NK cell GzmB was measured after 16–20 hr incubation. (A) Representative FACS plots are shown on the left and MFI of GzmB in NK cell population of biological duplicates is shown on the right. (B and C) Splenocytes were incubated with the indicated cytokine without stimulator splenocytes. (C) The concentration of the cytokines used were IFNα4 (200 U/ml), IFNβ (200 U/ml), IL-12 (20 ng/ml), IL-2 (200 U/ml), IL-6 (20 ng/ml), IL-15 (20 ng/ml), IL-18 (20 ng/ml). (D) IL-12Rβ2^-/-^ or IFNAR1^-/-^ splenocytes were mixed with cytokine receptor sufficient m157-Tg or control splenocytes. MFI = median fluorescence intensity. (E) Purified NK cells were cultured with m157-Tg or WT control MEF and analyzed as in (A). Data are representative of at least two independent experiments.

Since control of MCMV via cytotoxic NK cell effector function is critically dependent upon the pro-apoptotic activity of GzmB following degranulation [31], and we demonstrated that degranulation, *per se*, was not influenced by IFN-I or IL-12, we next assessed the ability of these cytokines to affect GzmB protein expression. Indeed, addition of either IFNβ or IL-12 to NK cells *in vitro* induced GzmB production, without affecting Ly49H levels (Fig 4A). The increase in GzmB MFI likely corresponds to increased protein levels of GzmB rather than a conformational change in GzmB such that it is more readily detectable as the NK cells show more cytotoxic function. However, it cannot be distinguished if the increased GzmB MFI reflects greater GzmB content per granule, greater number of GzmB+ granules, or a combination of both. IFNβ was more potent than IL-12 in inducing GzmB. Signaling through Ly49H itself with m157-Tg targets did not induce GzmB and did not substantially enhance GzmB levels induced by cytokines. Stimulation of GzmB production by cytokines is consistent with prior studies [31], but the role of IFN-I had not been extensively evaluated. Since IFNα and IFNβ could differentially affect GzmB production, we compared them directly. Both IFN-I subtypes induced GzmB in a dose-dependent fashion, but IFNβ was >30 times more potent in inducing GzmB than IFNα4 (Fig 4B). Simultaneous addition of IFNβ and IL-12 resulted in a synergistic effect on GzmB production and reached levels similar to those induced by IL-2 and IL-15 (Fig 4C). To determine if the requirements for IFNAR1 or IL-12Rβ2 were NK cell-intrinsic for GzmB production, splenocytes deficient in either of these receptors were stimulated with cytokine and WT stimulator cells. In terms of GzmB protein production, NK cells deficient in IL-12Rβ2 did not respond to IL-12, but still responded to IFNβ whereas IFNAR1^-/-^ NK cells did not respond to IFNβ, but responded to IL-12 (Fig 4D). This NK cell-intrinsic response was confirmed by stimulating purified NK cells with MEFs and IFNβ or IL-12 (Fig 4E). Intriguingly, the IFNβ-induced GzmB accumulation was greater in the Ly49H^+^ compared to the Ly49H^-^ NK cells, whereas in response to IL-12, GzmB accumulation was more pronounced in the presence of m157 (S2A Fig). Taken together, these data suggest that both Ly49H and either IFNβ or IL-12 are intrinsically necessary for efficient cytotoxicity *in vivo*.

### IFN-I and IL-12 are redundantly required for effective granzyme B and perforin accumulation in NK cells

**Fig 5:**
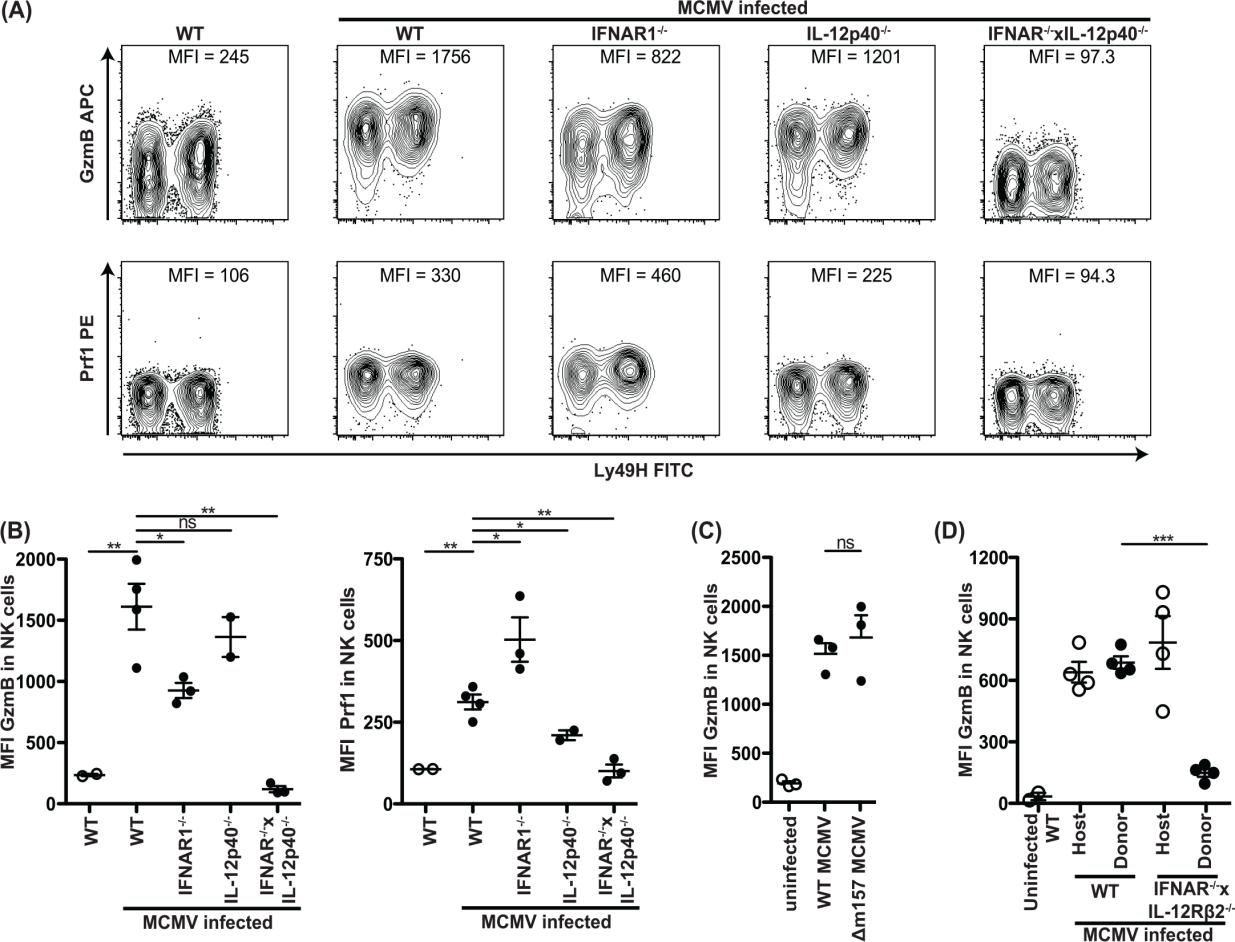
IFN-I or IL-12 are required for GzmB and Prf1 induction in NK cells in a cell intrinsic manner during MCMV infection. (A and B) Mice deficient in IFNAR1 and/or IL-12p40 were infected with WT MCMV at 5000 PFU/mouse. After 3 days, splenocytes were harvested and analyzed for GzmB and Prf1 expression as indicated. Representative contour plots of individual mice are shown in (A). The median fluorescence intensity (MFI) is indicated for each plot and the cumulative data for one experiment is shown in (B). (C) GzmB levels of WT mice infected with 1x10^5^ PFU WT or Δm157 MCMV 2 days after infection. (D) 40x10^6^ WT or IFNAR1^-/-^xIL-12Rβ2^-/-^ splenocytes were adoptively transferred to congenic host that was simultaneously infected with 1x10^5^ PFU MCMV/mouse. GzmB levels 2 days post-infection in host and transferred NK cells is depicted. Data are representative of three to four independent experiments with individual points representing a single mouse.

Interestingly, at three days following MCMV infection, we found that levels of GzmB and Prf1 in splenic NK cells examined *ex vivo* were significantly elevated in mice with deficiency in either IFNAR1 or IL-12p40 (Fig 5A and 5B). Upregulation of GzmB appeared to be equivalent in both Ly49H^+^ and Ly49H^-^ cells and did not require the presence of m157-expressing virus at 40 hours after infection (Fig 5C). IFNAR1^–/–^mice demonstrated a less robust increase in the expression of GzmB, as compared to WT and IL-12p40-deficient mice which displayed equivalent levels. Prf1 production was increased in IFNAR1^–/–^mice while IL-12p40^–/–^mice displayed decreased levels of Prf1 compared to WT, suggesting that GzmB and Prf1 are differentially regulated. Importantly, double cytokine-deficient mice did not display enhanced GzmB nor Prf1 protein levels following infection (Fig 5A and 5B). Indeed, the GzmB levels were even below those observed in uninfected WT mice, suggesting that both IL-12 and IFN-I contribute to production of small amounts of GzmB in naïve mice. For Prf1 this effect was not clearly observable, which may be due to the less robust Prf1 changes or detection. WT MCMV induced elevated levels of GzmB in Ly49H^+^ NK cells compared to their Ly49H^-^ counterparts, similar to what was observed *in vitro* (S2 Fig). Importantly, this effect was also observed for Δm157 MCMV (S2B Fig), further indicating that signaling through Ly49H is not required for enhanced GzmB production in Ly49H^+^ NK cells during MCMV infection. Regardless, both IL-12 and IFN-I redundantly contribute to enhanced GzmB and Prf1 protein expression during MCMV infection.

To confirm that NK cell-intrinsic signaling by cytokines was required, we adoptively transferred WT or IFNAR1^-/-^xIL-12Rβ2^-/-^ splenocytes into congenic WT hosts that were simultaneously infected with MCMV. We observed no increase in GzmB levels in the double-deficient donor NK cells whereas WT donor control and WT host control NK cells produced the expected increased levels of GzmB (Fig 5D). These results indicate that the enhancement of GzmB protein expression by IFN-I or IL-12 signaling is NK cell-intrinsic during MCMV-infection.

### IFN-I signaling is required for optimal Ly49H-dependent cytotoxicity *in vivo*

**Fig 6:**
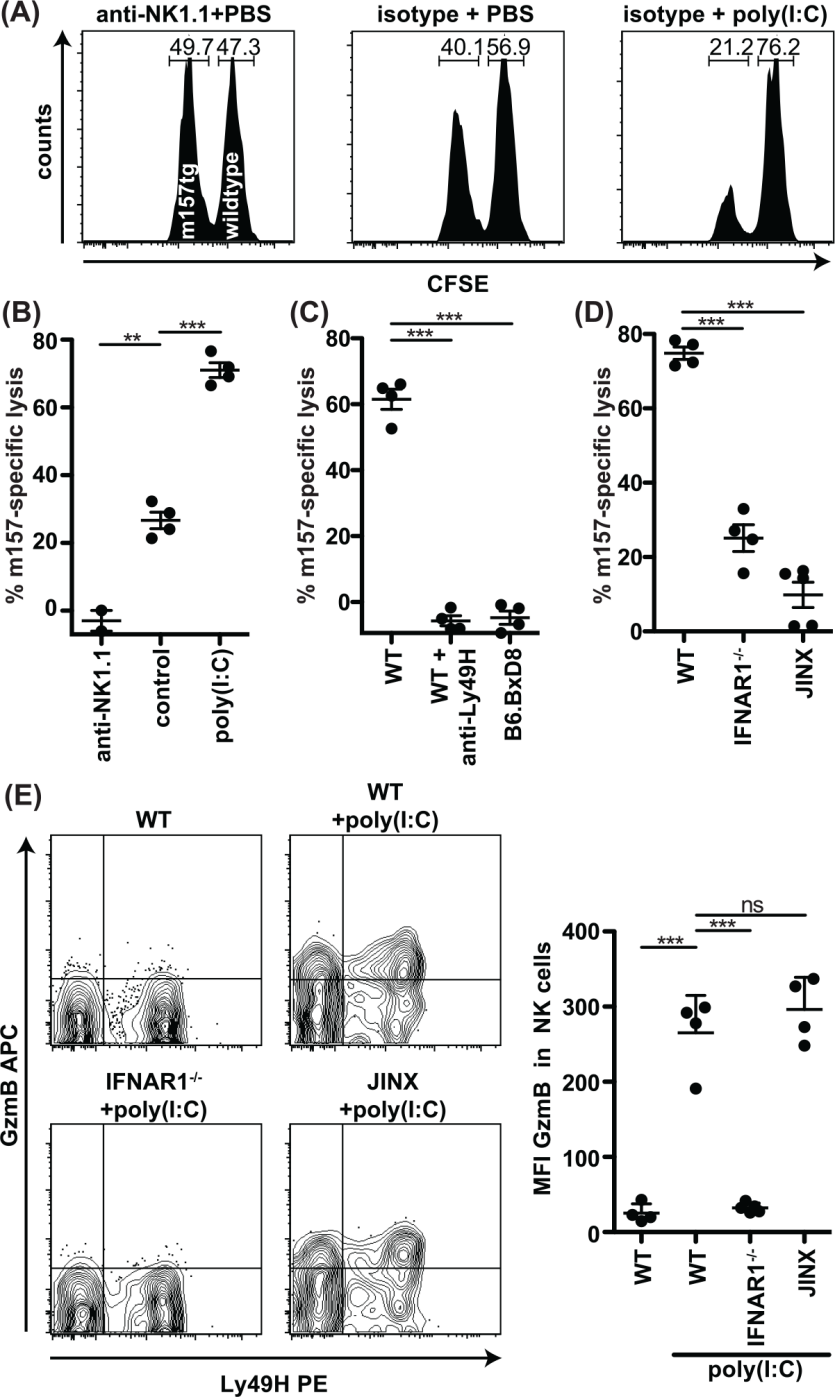
m157-specific cytotoxicity *in vivo* is greatly enhanced by poly(I:C) and dependent on IFN-I and degranulation. (A-D) Mice were i.p. injected with 100 μg poly(I:C), PBS, 200 μg anti-NK1.1, 200 μg anti-Ly49H and/or isotype control. After 16 hours 2x10^6^ differentially CFSE-labeled splenocytes were injected intravenously. Three hours after adoptive transfer splenocytes were analyzed by flow cytometry. (A) Representative histograms showing splenic CD19^+^CFSE^+^ cells. Numbers indicate percentage of m157-Tg CFSE^low^ and WT CFSE^high^ cells. (B-D) Specific lysis of m157-Tg targets. (E) Mice were injected IP with 100 ug poly(I:C) or PBS. After 16 hours the spleens were harvested and NK cells were analyzed for GzmB. Data are representative of two to three independent experiments with individual points representing a single mouse.

To directly determine the requirement for both Ly49H and IFN-I activation for cytotoxicity *in vivo*, we determined the *in vivo* cytotoxic elimination of m157-expressing cells (Fig 6A). Following injection of equal numbers of congenic m157-Tg and WT splenocytes differentially labeled with CFSE into control-treated mice, we found there were low levels (15–35%) of m157-specific elimination (Fig 6A and 6B), consistent with previous findings on m157-specific rejection under steady-state conditions [37, 38]. Anti-NK1.1 depletion abrogated m157-specific elimination of target cells, confirming NK cell dependence. However, mice pretreated with poly(I:C) exhibited 3-fold higher levels of m157-specific rejection as compared to control-treated mice (Fig 6A and 6B). Blockade of Ly49H prevented m157-expressing target elimination in poly(I:C)-treated mice, confirming the role of Ly49H (Fig 6C). Similar results were obtained in B6.BxD8 mice specifically lacking the Ly49H receptor. Specific elimination was also abrogated in Jinx mice (Fig 6D), indicating that m157-expressing target elimination *in vivo* in poly(I:C)-treated mice is due to cytotoxicity. The m157-specific killing was 3-fold lower in IFNAR1^-/-^ mice treated with poly(I:C) as compared to WT control mice, showing IFN-I signaling affects m157-specific rejection (Fig 6D). This defective cytotoxic capacity in IFNAR1^-/-^ mice correlated with a lack of increased GzmB production in response to poly(I:C) (Fig 6E). Conversely, Jinx mice failed to eliminate m157-expressing targets (Fig 6D) even though GzmB levels were similar to WT mice (Fig 6E), highlighting the requirement of both degranulation and cytokine activation for optimal Ly49H-dependent cytotoxicity *in vivo*.

### m157-specific cytotoxicity during MCMV infection *in vivo* depends on IL-12 and IFN-I

**Fig 7:**
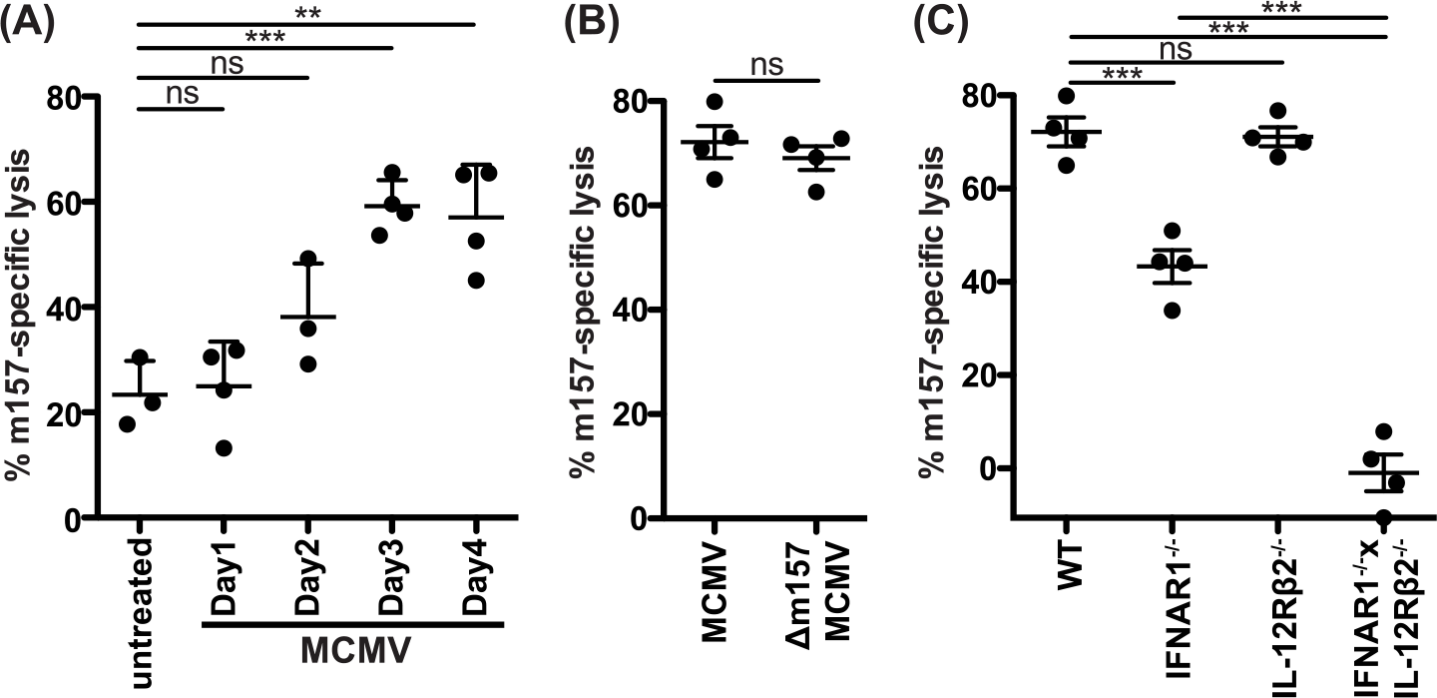
IL-12 or IFN-I are required for m157-specfic cytotoxicity *in vivo* during MCMV infection. Mice were i.p. injected with MCMV (10000 PFU/mouse) and at the indicated timepoint after infection an *in vivo* cytotoxicity assay was performed as in Fig 6. **(A)** m157-specific rejection was analyzed at days 1–4 after MCMV infection. **(B)** Mice were infected with WT or Δm157 MCMV for 3 days, after which m157-specific rejection was analyzed. **(C)** m157-specific rejection was measured in mice deficient in IFNAR1, IL-12Rβ2, or both was analyzed at 3 days post-infection. Data are representative of three to four independent experiments with individual points representing a single mouse.

To directly assess the role of cytotoxicity during MCMV infections, we performed rejection assays in infected mice. During the first day post-infection, *in vivo* cytotoxicity of m157-Tg targets was similar to untreated controls while it increased on day 2 and plateaued at days 3 and 4 (Fig 7A). At day 2 after infection, GzmB and perforin responses in the NK cells peaked (S3A Fig and [31]), but the cytotoxic capacity was only modestly but not significantly increased. However, at this time the number of detectable NK cells in the spleen dramatically drops to approximately 30% of steady-state levels [39], which may explain the discrepancy of enhanced GzmB and perforin production in NK cells with only modestly increased *in vivo* cytotoxic capacity. At day 3–4 after infection, GzmB levels decreased (S3A Fig) but the number of detectable NK cells increased and the net result was more potent m157-specific target clearance. Despite a requirement for m157 expression on the target, there was otherwise no overt role for m157 in potentiating NK cell cytotoxicity during MCMV infection, as m157-specific rejection of targets was similar at day 3 in mice infected with Δm157 or WT MCMV (Fig 7B). These data show that NK cell cytotoxicity increases during the first days after MCMV and plateaus at day 3, which reflects the capacity of NK cell mediated control of MCMV during this time.

Finally, we found mice deficient in IFNAR1 were substantially hampered in their cytolytic capacity during MCMV infection (Fig 7C). These mice showed a 40% reduction in m157-specific killing, but the remaining cytotoxic capacity was still sufficient for Δm157 selection, though somewhat incomplete (Fig 2B). Interestingly, IL-12 alone played no detectable role in cytotoxicity *in vivo* as IL-12Rβ2^-/-^ mice showed similar levels of m157-specific rejection. However in mice deficient in both IFNAR1 and IL-12Rβ2, m157-specific killing was reduced to below baseline WT levels (Fig 7C). Moreover, uninfected IFNAR1^-/-^xIL-12Rβ2^-/-^ mice showed reduced baseline m157-specific target rejection despite normal maturation and functional capacity of the IFNAR1^-/-^xIL-12Rβ2^-/-^ NK cells (S3B–S3E Fig), showing a role for cytokine activation even under steady-state conditions. Together, these results closely mirror the results on m157-dependent MCMV selection as well as GzmB production (Figs 2E, 3A and 4A), indicating that both IFN-I and IL-12 contribute to NK cell cytotoxicity during MCMV infections.

## Discussion

Herein we used an *in vivo* viral competition assay to specifically demonstrate that Ly49H-mediated MCMV control is predominately due to cytotoxicity that is enhanced by cytokine signaling, independent of IFNγ. All strains of mice tested here with defects in cytotoxicity, including granule formation (Lyst), exocytosis (Jinx), or perforin, showed the same effect, i.e., an inability of Ly49H^+^ NK cells to control MCMV, as evidenced by an absence of Δm157 MCMV selection. Our findings are independent of overall viral titers, providing unequivocal evidence for cytotoxicity despite potential confounding effects.

A recent report described that both Ly49H-m157 engagement and IFNγ production were important in NK-mediated control of MCMV [20]. Interestingly, mortality did not correlate well with splenic titers in prior studies that have examined mice globally deficient in IFNγ, suggesting that the cytokine likely potentiates other critical aspects of the innate immune response. For example, Fodil *et al* demonstrated a marked effect on survival with only a mild (1-log) increase in splenic titers of WT MCMV if Ly49H was transgenically expressed in a mouse strain deficient for IFNγ production [20]. We have confirmed these overall WT MCMV titer differences in our studies within both the splenic and hepatic compartments of IFNγ^-/-^ mice. However, our data suggest that global defects can affect Ly49H-independent viral control, suggesting that the role of IFNγ in antiviral control by Ly49H^+^ NK cells can be difficult to resolve when studying mice globally lacking immune molecules and assessing overall viral titers [18, 19].

Interestingly, Ly49H-dependent degranulation occurred in naïve mouse NK cells *in vitro*, reminiscent of activation receptor-induced granule exocytosis of human NK cells in an LFA-1-dependent manner in an *in vitro* system that limited the number of receptor and ligand pairs. The combination of engagement of Fc receptor and LFA-1 by ICAM on insect cells facilitated human NK cell degranulation [14] and triggering of multiple activation receptors was shown to further increase human NK cell degranulation [40]. Of note is that our *in vitro* assays utilized syngeneic splenocytes as stimulators differing from control splenocytes only in the transgenic expression of m157. The stimulator cells therefore expressed the steady state repertoire of the relevant adhesion molecules as well as activation and inhibitory ligands, which together contributed to degranulation. Nonetheless, our studies indicate that degranulation was insufficient for effective target killing and provide a cautionary note that assessment of degranulation by CD107a may not be a good correlate for cytotoxicity under certain conditions.

Our studies indicate that effective NK cell cytotoxicity requires other signals, in addition to Ly49H triggering. The *in vivo* viral competition assay demonstrated a requirement for either IL-12 or IFN-I acting directly on NK cells to promote activation receptor-mediated killing of MCMV-infected cells. In the presence of either IL-12 or IFN-I alone, Ly49H-dependent control appeared to occur, despite overall increases in viral titers, likely due to the pleiotropic effects of these cytokines on the immune response. Interestingly, the effect of IFN-I was much greater than that of IL-12, but it was only when both were genetically absent and/or neutralized, that Ly49H-dependent control was markedly affected, as shown by significant effects on Δm157 MCMV selection. Regardless, both cytokines can independently contribute to Ly49H-mediated cytotoxicity via a mechanism involving upregulation of GzmB protein expression, markedly expanding prior observations that IL-15 can also induce this effect [30, 31].

Our results showing an important role for IFN-I and IL-12 on Ly49H-dependent viral control was unexpected as IL-15 is a potent stimulator of GzmB protein production and IL-15 is produced during MCMV infections [31, 41]. Prior studies suggested a role for cross-presentation of IL-15 by dendritic cells as a critical component in NK cell responses during viral infections [30]. In the current study, we did not examine the role of IL-15 because mice lacking IL-15 or any component of the IL-15 receptor lack NK cells [42]. However, here we indirectly addressed the role of IL-15 priming of NK cells by DCs by utilizing Batf3-deficient mice [43] that fail to develop CD8α DC subsets. We observed that Batf3-dependent DCs were not required for the Ly49H-dependent cytotoxicity observed during MCMV infection. Although IL-15 should act directly on NK cells and is thought to be downstream of IL-12 and IFN-I stimulation of DCs, we found that the IL-12 and IFN-I effects studied here were due to direct effects of these cytokines on the NK cells themselves. Therefore, our current studies showed that IL-12 and IFN-I had profound effects directly on NK cells, suggesting that IL-15 could account for the residual IL-12- and IFN-I-independent effects on Ly49H-mediated MCMV control, or that IL-15 acts together with IL-12 and IFN-I in a more complex manner.

Our findings here also markedly extend prior studies on the role of both IFN-I and IL-12 in affecting NK cell responses during MCMV infection. Recently, both IFN-I and IL-12 were shown to induce the high-affinity IL-2 receptor (CD25) on NK cells via a STAT4-dependent mechanism [44]. However, the consequence of these cytokines on CD25 expression was studied only for NK cell proliferation during MCMV infection. The role of proliferation, *per se*, in MCMV control is unclear because Ly49H-specific proliferation is detectable after Ly49H-dependent MCMV control had already occurred [39]. By contrast, we report here that both cytokines are required for Ly49H activation receptor-dependent killing and are critical for MCMV control.

The roles of IL-12 and IFN-I during MCMV infection also have been extensively studied by others [23]. Initially, it was reported that NK cell-dependent IFNγ production required an IL-12-mediated pathway while cytotoxicity was enhanced through IFN-I signaling during MCMV infection. Another role of IFN-I is to promote chemotaxis of NK cells to virally infected livers [45] but Ly49H-mediated effects are generally not observed in the liver [3]. More recent characterization of both IL-12 and IFN-I dependent pathways intrinsic to NK cells during MCMV infection has demonstrated non-redundant effects on cytotoxicity, IFNγ production, and survival [41]. IFN-I signaling clearly promoted NK cell cytotoxicity but it was not previously clear if these effects were Ly49H-dependent or Ly49H-independent. Here we report that IL-12 and IFN-I pathways act redundantly to enhance Ly49H-dependent cytotoxicity.

IFNα was previously reported to only modestly induce GzmB in NK cells [31]. However, IFNα and IFNβ are both induced during acute MCMV infection with similar kinetics [46]. Here we show IFNβ is much more potent than IFNα in inducing GzmB in NK cells, consistent with reported increased immune responses to IFNβ as compared to IFNα [47]. IFNβ only requires the IFNAR1 subunit, whereas IFNα requires both the IFNAR1 and IFNAR2 subunits [48], which may provide a molecular explanation to the observed differences between both IFN-I molecules. Regardless, our studies suggest that IFNβ may be the primary IFN-I in mediating NK cell mediated viral control during acute MCMV infection, but due to the large number of IFN-I molecules, additional work will be needed to determine if a specific IFN-I molecule is critical for Ly49H-dependent control.

Finally, there are clear parallels between our work and cytokine-involvement in T cell responses, as cytokines have been described to act as a “third” signal, in addition to T-cell receptor and co-stimulatory molecule engagement during T-cell priming as well as proliferation and survival [49]. This third signal has been found to be essential for priming of CD8^+^ T cells as well as enhancing their effector functions [50]. Analogous to the effect of cytokines on T-cell expansion, IL-12 and IL-15 have been described to play a central role in proliferation and survival of NK cells [51, 52]. Our data now show that other cytokines provide crucial signals to NK cells for activation receptor-mediated cytolytic functions, beyond that of NK cell proliferation and survival.

In conclusion and based on results reported here, we propose the following model, where NK cells require two signals for efficient cytolytic effector function. Signaling through a cytokine receptor on the NK cell itself, which is provided by IL-12 or IFN-I in the case of MCMV infection, results in the accumulation of cytotoxic effector molecules, including GzmB. Activation receptor recognition of target cells through ligands, such as m157, signals NK cell release of cytotoxic cargo, ultimately leading to killing of the (virus-infected) target cell and MCMV control. In the absence of cytokine stimulation, Ly49H activation may occur, resulting in granule exocytosis, but the NK cells will be impotent killers because cytotoxic effector molecules, such as GzmB, are poorly expressed. Conversely, absence of activation receptor stimulation leads to poor NK cell control. Thus, NK cells require two intrinsic signals to mediate effective cytotoxic control of viral infection.

## Materials and Methods

### Mice

B6 (and congenic Ly5.2 B6) mice were purchased from either the National Cancer Institute (Fredrick, MD) or Charles River Laboratories (Wilmington, MA). The following strains were purchased from Jackson Laboratory: IL-12Rβ2-/- (003248), IL-12p40-/- (002693), Prf1-/- (002407), IFNγ-/- (002287), IFNγR-/- (003288), IL-18-/- (004130), Lyst (000629), and RAG1-/- (002216). Jinx mice, harboring a mutation in UNC13D, and TRIF-/- mice, were both provided by B. Beutler (Scripps Research Institute, La Jolla, CA). MyD88-/- mice were provided by S. Akira (Osaka University, Osaka, Japan) or purchased through the Jackson Laboratory (009088). DAP12 KI mice were provided by E. Vivier (CNRS-INSERM-Universite de la Mediterranee, France). DAP12-/- and DAP10-/-DAP12-/- [53] mice were provided by T. Takai. DAP10-/- [54] mice were provided by M. Colonna. Batf3^-/-^ [43] mice were provided by K. Murphy. IFNAR1-/- [55], m157-Tg [36] and Ly49H-deficient B6.BxD8 [56] mice were generated and maintained in our laboratory. The Speed Congenics Facility of the Rheumatic Diseases Core Center at Washington University assisted in backcrossing mice to the B6 background using a marker-assisted approach. Standard breeding strategies were used to generate double deficient MyD88-/-TRIF-/- mice, IL-12p40-/-IFNαβR-/- mice, and IL-12Rβ2-/-IFNαβR-/- mice. All mice used were generated on or backcrossed to the C57BL/6 (B6) genetic background. All mice were used in accordance with institutional guidelines for animal experimentation.

### Viral infection and generation of Δm157-MCMV

The salivary gland propagated MCMV stocks were generated from purified and sequenced clones. Virus was inoculated via the intraperitoneal (IP) route in a total volume of 200μl PBS. The complete genomic sequence for the strain designated WT1 was previously published [57]. To generate an escape virus deficient in m157 expression, WT1 MCMV was passaged in a T cell-deficient host (TCRbd-/-; Jackson Laboratory, 002122) for 3 weeks, followed by plaque purification of spleen-derived virus and subsequent assessment of Ly49H-engagement as described previously [10]. The entire genome of the Δm157-MCMV was sequenced (HiSeq, Illumina) and a single G to T nucleotide substitution at position 502 of m157 (numbering relative to translational start codon) was identified as the only deviation in the 230kb sequence compared with WT1, consistent with our prior studies indicating the stability of MCMV and the role of Ly49H in specifically selecting mutations in m157 [10, 57]. The base substitution results in a premature stop codon (GAA to TAA) within the m157 ORF. An *in vitro LacZ*-based Ly49H-reporter assay [5] confirmed that the Δm157-MCMV lacked m157 expression and was unable to engage the Ly49H receptor. A multi-step growth curve [58] was performed on NIH 3T12 (ATCC CCL-164) fibroblasts (multiplicity of infection [MOI] = 0.1). Viral genome copies quantified from cell lysates and culture supernatants demonstrated that replication of Δm157-MCMV was equivalent to WT MCMV (S1J Fig). To generate an inoculum containing 10% Δm157-MCMV, 2000 PFU of Δm157-MCMV was mixed with 18000 PFU of WT1 virus, both of salivary gland origin.

### Viral genome quantification

Genomic DNA was prepared from mouse tissues, cell lysates, and culture supernatants using the Puregene extraction kit (Qiagen) following manufacturer recommendations. DNA was adjusted to achieve a concentration between 20–50ng/μl. Each qPCR reaction consisted of 5μl of TaqMan Universal PCR Master Mix, No AmpErase UNG (Life Technologies, #4324018), 2μl of organ DNA, 0.5μl of a 20X primer/probe mixture (PrimeTime qPCR Assay, IDT) with sequences as described in S1 Table, and 2.5μl water. This is done for both IE1 and actin in separate reactions. Amplification was performed on the AppliedBiosystems StepOne Plus qPCR instrument (Life Technologies). Each reaction was run in duplicate and quantified against a standard curve to ensure linearity, sensitivity, and enable comparison between independent runs. The standard curve efficiencies were consistently near 100%. Data were analyzed by first generating a ratio of MCMV IE DNA copies/murine actin DNA copies. As actin appears to be present in about a 1000-fold excess over IE1, multiplying the resulting ratio by 1000 closely reflects PFU values obtained by standard plaque assays.

### Viral escape frequency determination

To calculate the frequency of escape virus (Δm157-MCMV) following infection, genomic DNA was prepared as for viral genome quantification. The primer/probe mixtures (S1 Table) contained Taqman probes for both WT and m157-deficient alleles in a single tube, differentiated via the dye conjugate (Custom TaqMan SNP Genotyping Assay, Life Technologies, #4332075). To validate the specificity and dynamic range of the Taqman-based genotyping assay, we initially determined the ratio of SNP-specific product to WT product using predetermined mixtures of 1%, 5%, 10%, 25%, 50%, 75%, and 100% Δm157-MCMV. The ΔΔCT (cycle threshold) was calculated as a difference between the CT of the 100% Δm157-MCMV sample and the individual reference samples. A second-order polynomial (quadratic) function to describe the slope of the line through these points allowed accurate determination of Δm157-MCMV frequencies between 5% and 75% (S1K Fig). In validation assays, the percent of Δm157 that was greater than 75% could not be quantified due to cross-reactivity with the Taqman probe for WT MCMV. Therefore 75% was considered the limit of quantification (L).

### Antibody depletions

Purified 3D10 (α-Ly49H), PK136 (α-NK1.1), and MAR (isotype controls) were obtained from hybridomas purified by the Rheumatic Diseases Core Center Protein Purification and Production Facility. MAR1-5A3 (α-IFNAR1) [59] and GIR-208 (IFNAR1 isotype control, [60] were previously described. Antibodies was injected IP at a dose of 200μg per mouse 24hrs (α-Ly49H) or 48 hrs (α-NK1.1) prior to infection, or 2mg (α-IFNAR1) per mouse 24 hrs prior to infection.

### Reagents and cell preparation

Fluorescent-labeled antibodies used were, anti-NK1.1 (clone PK136), anti-NKp46 (29A1.4), anti-CD3 (145-2C11), anti-CD19 (eBio1D3), anti-CD45.1 (A20), anti-CD45.2(104), anti-CD107a (eBio1D4B), anti-perforin (eBioOMAK-D) were purchased from Affymetrix, anti-granzyme B (GB12) and isotype control were purchased from Life Technologies, and biotinylated anti-Ly49H (clone 3D10) was produced as described earlier [36]. IFNα4 and IFNβ were purchased from PBL Assay Science, IL-6, IL-12, IL-15 from Peprotech, IL-18 from MBL, and IL-2 was produced in-house. NK cells were purified from RAG1^-/-^ splenocytes to a purity >95% using CD49b microbeads (Miltenyi Biotec) according to manufacturer’s instructions. m157-Tg murine embryonic fibroblasts were isolated from day 11.5–13.5 embryos, WT embryos from the same pregnant female served as *in utero* controls.

### Flow cytometry

All cells were first stained with fixable viability dye (Affymetrix), followed by surface staining with directly conjugated antibodies or in two steps using streptavidin-PE (Becton Dickinson) in 2.4G2 hybridoma supernatant to block Fc receptors and washed with PBS with 0.5% BSA and 0.02% Sodium Azide (PBA). Subsequently, samples were fixed and stained intracellularly using Cytofix/Cytoperm kit according to manufacturer’s protocol (BD Biosciences). Samples were analyzed by flow cytometry using FACSCanto (BD Biosciences) and FACScan with DxP6 upgrade (Cytek). Data was analyzed using FlowJo software (Tree Star). NK cells were defined as Viability-NK1.1^+^CD3^-^CD19^-^.

### *In vitro* stimulation assays

Freshly isolated splenocytes were co-cultured with congenic CD45.1/2 or CFSE-labeled m157-Tg or littermate control splenocytes at a 1:1 ratio in the presence or absence of cytokine at the concentration indicated. For degranulation assays anti-CD107a and monensin (Affymetrix) was added after 1 hour and the reaction was stopped 5–8 hours later by the addition of PBA. For GzmB staining, samples were stained after 16–20 hours after initiation of co-culture. For stimulation assays with pure NK cells, NK cells were co-cultured with MEF at a 1:1 ratio.

### *In vivo* cytotoxicity assay

Target splenocytes isolated from Ly5.1xLy5.2 m157-Tg animals were labeled with 1μM CFSE (Life technologies) and Ly5.1xLy5.2 WT littermate controls were labeled with 5μM CFSE. CFSE^low^ and CFSE^high^ target cells were mixed at a 1:1 ratio and 2-3x10^6^ target cells were injected i.v. into naïve or treated mice as described. After 3 hours challenge splenocytes were harvested and stained. The ratio of CFSE^low^ and CFSE^high^ viable CD19^+^ cells was determined by flow cytometry. Target cell rejection was calculated using the formula [(1−(Ratio(CFSE^low^:CFSE^high^)_sample_/Ratio(CFSE^low^:CFSE^high^)_control_)) × 100]. Average of 2–3 NK1.1-depleted mice and input mixture served as control.

### Statistical analysis

Statistical analysis was performed using Prism (GraphPad software). Unpaired, two-tailed Student's *t-*tests were used to determine statistically significant differences between experimental groups. Error bars in figures represent the SEM.

### Ethics statement

This study was carried out in strict accordance with the recommendations in the Guide for the Care and Use of Laboratory Animals of the National Institutes of Health. The protocol was approved by the Animal Studies Committee at Washington University School of Medicine under animal protocol 20130049A2.

## Supporting Information

S1 Fig Selection of m157-deficient virus during a dual infection assay is dependent upon the NK cell Ly49H activation receptor. **(A,C-G,I)** Viral titers and **(B,H)** viral escape (Δm157) frequency determined five days after a mixed MCMV infection with 2e4 PFU/mouse in spleen **(A-G)** and liver **(H,I)**. In **(B)** and **(C)**, 1e6 PFU/mouse was inoculated as indicated. **(A)** α-NK1.1 (or isotype control) was administered 48hrs prior to infection. **(G)** Statistical analysis performed by comparing values from WT mice with genetically modified or antibody-treated mice. For all panels, individual points represent a single mouse. L = Limit of quantification (75%). I = Input frequency (10%). **(J)** Multi-step growth curve of NIH 3T12 fibroblasts initially infected with a multiplicity of infection (MOI) of 0.1 with the indicated virus stocks. Virus was isolated from both supernatant and cell lysates and quantified via qPCR. **(K)** Assessment of known mixtures of WT and m157-deficient virus using a Taqman genotyping served as standards to quantify results. A trendline is depicted with the quadratic expression that defines the slope and the indicated R-squared value. The ΔΔCT refers to the log-2 transformed qPCR cycle threshold (CT) of the Δm157 Taqman probe subtracted from the WT probe, with 100% Δm157 as the comparator (*i*.*e*. for 100% Δm157, ΔΔCT = 0).
(EPS)

S2 Fig Increased GzmB production in Ly49H^+^ NK cells independent of Ly49H triggering. **(A)** GzmB levels in Ly49H^+^ versus Ly49H^-^ NK cells stimulated *in vitro* as in Fig 4A. **(B)** GzmB levels in Ly49H^+^ versus Ly49H^-^ splenic NK cells after MCMV infection *in vivo* as in Fig 5C.
(EPS)

S3 Fig IFNAR1^-/-^xIL12Rβ2^-/-^ NK cells have reduced cytotoxic activity at steady state, but are fully functional in degranulation and GzmB production. **(A)** GzmB in NK and percentage of NK cells in mice treated as in Fig 5A. **(B)** m157-specific rejection as in Fig 7. **(C)** Expression of CD27 and CD11b on WT versus double deficient NK cells. **(D)** GzmB response in NK cells to cytokine stimulation as in Fig 4. **(E)** Degranulation in NK cells in response to m157 and cytokine stimulation as in Fig 3.
(EPS)

S1 Table Primer and probe sequences for quantitative PCR amplifications. (TIF)
