# Supplementary figures and images for: Dual Requirement of Cytokine and Activation Receptor Triggering for Cytotoxic Control of Murine Cytomegalovirus by NK Cells
Source: PLoS Pathog. 2015 Dec 31;11(12):e1005323. doi: 10.1371/journal.ppat.1005323 (PMC4697817; doi:10.1371/journal.ppat.1005323)

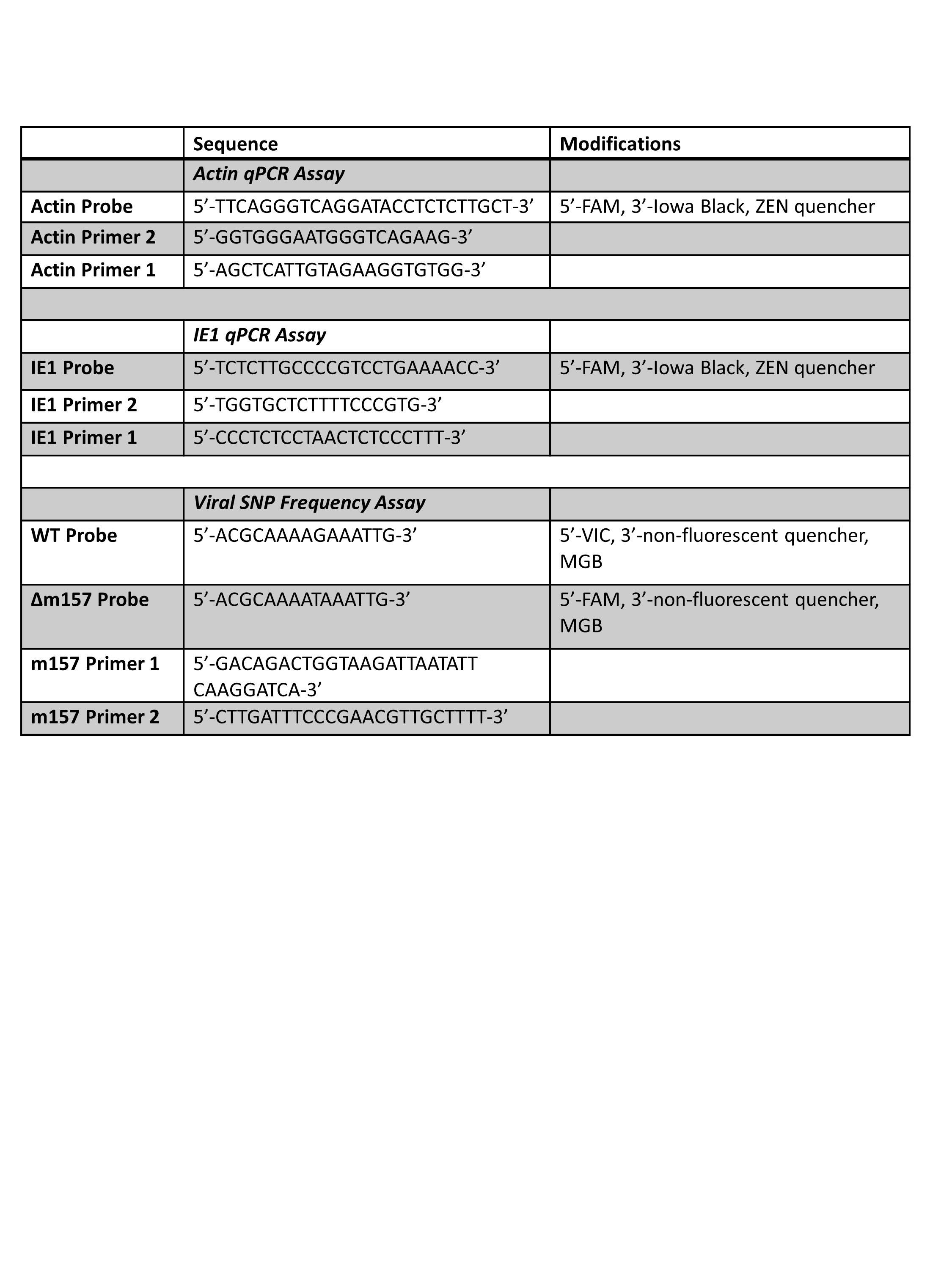

Supplement: S1 Table — (TIF) [file ppat.1005323.s004.tif]
